# *bla*_VIM_- and *bla*_OXA_-mediated carbapenem resistance among *Acinetobacter baumannii* and *Pseudomonas aeruginosa* isolates from the Mulago hospital intensive care unit in Kampala, Uganda

**DOI:** 10.1186/s12879-019-4510-5

**Published:** 2019-10-16

**Authors:** Dickson Aruhomukama, Christine F. Najjuka, Henry Kajumbula, Moses Okee, Gerald Mboowa, Ivan Sserwadda, Richard Mayanja, Moses L. Joloba, David P. Kateete

**Affiliations:** 1 Department of Medical Microbiology, College of Health Sciences, Makerere University, Kampala, Uganda; 2 Department of Immunology & Molecular Biology, College of Health Sciences, Makerere University, Kampala, Uganda

**Keywords:** Carbapenem resistant *Acinetobacter*, Carbapenem resistant *Pseudomonas aeruginosa*, Carbapenemase genes, Class 1 integrons, Conjugation, Intensive care unit, ICU, Mulago hospital, Uganda

## Abstract

**Background:**

Between January 2015 and July 2017, we investigated the frequency of carbapenem resistant *Acinetobacter baumannii* (CRAB) and carbapenem resistant *Pseudomonas aeruginosa* (CRPA) at the Mulago Hospital intensive care unit (ICU) in Kampala, Uganda. Carbapenemase production and carbapenemase gene carriage among CRAB and CRPA were determined; mobility potential of carbapenemase genes via horizontal gene transfer processes was also studied.

**Methods:**

Clinical specimens from 9269 patients were processed for isolation of CRAB and CRPA. Drug susceptibility testing was performed with the disk diffusion method. Carriage of carbapenemase genes and class 1 integrons was determined by PCR. Conjugation experiments that involved *bla*_VIM_ positive CRAB/CRPA (donors) and sodium azide resistant *Escherichia coli* J53 (recipient) were performed.

**Results:**

The 9269 specimens processed yielded 1077 and 488 isolates of *Acinetobacter baumannii* and *Pseudomonas aeruginosa*, respectively. Of these, 2.7% (29/1077) and 7.4% (36/488) were confirmed to be CRAB and CRPA respectively, but 46 were available for analysis (21 CRAB and 25 CRPA). Majority of specimens yielding CRAB and CRPA were from the ICU (78%) while 20 and 2% were from the ENT (Ear Nose & Throat) Department and the Burns Unit, respectively. Carbapenemase assays performed with the MHT assay showed that 40 and 33% of CRPA and CRAB isolates respectively, were carbapenemase producers. Also, 72 and 48% of CRPA and CRAB isolates respectively, were metallo-beta-lactamase producers. All the carbapenemase producing isolates were multidrug resistant but susceptible to colistin. *bla*_VIM_ was the most prevalent carbapenemase gene, and it was detected in all CRAB and CRPA isolates while *bla*_OXA-23_ and *bla*_OXA-24_ were detected in 29 and 24% of CRAB isolates, respectively. Co-carriage of *bla*_OXA-23_ and *bla*_OXA-24_ occurred in 14% of CRAB isolates. Moreover, 63% of the study isolates carried class 1 integrons; of these 31% successfully transferred *bla*_VIM_ to *E. coli* J53.

**Conclusions:**

CRAB and CRPA prevalence at the Mulago Hospital ICU is relatively low but carbapenemase genes especially *bla*_VIM_ and *bla*_OXA-23_ are prevalent among them. This requires strengthening of infection control practices to curb selection and transmission of these strains in the hospital.

## Background

Carbapenems are a highly potent class of antibiotics used to treat infections caused by multidrug resistant (MDR) Gram negative bacteria. However, the effectiveness of these antibiotics is currently being threatened by the emergence of carbapenem resistant bacteria [1, 2]. Carbapenemases are the main mechanism by which resistance to carbapenems occurs [1, 3–5], and they belong to three of the four β-lactamase classes A, B and D [1–5]. Class D carbapenemases are the OXA-β-lactamases [4], further subdivided into various sub-groups mainly *bla*_OXA-23_, *bla*_OXA-24/40_, *bla*_OXA-58_, *bla*_OXA-48_, *bla*_OXA-51_ and *bla*_OXA-143_ [1, 4]. These OXA-type β-lactamases occur widely in *Acinetobacter* with the most abundant being *bla*_OXA-51_, which is chromosomally encoded hence intrinsic to these species but it may confer resistance to carbapenems when its expression is up-regulated by genetic re-organization [1, 4, 6]. Class B carbapenemases are also known as the metallo-β-lactamases (MBLs) [1, 3]; they are mostly encoded by integron-borne mobile gene cassettes and hence, they are transferable amongst various bacteria via horizontal gene transfer mechanisms notably conjugation [3]. Class A carbapenemases include the *Klebsiella pneumoniae* carbapenemase (KPC) family that can be plasmid encoded or chromosomal [2, 5].

The hospital environment, mostly the intensive care units (ICUs), is a hotspot of antimicrobial resistance and source of MDR bacterial infections [7], particularly infections involving *Acinetobacter* species and *Pseudomonas aeruginosa*. The aim of this study was to determine the prevalence of carbapanem resistant *Acinetobacter baumannii* (CRAB) and carbapenem resistant *Pseudomonas aeruginosa* (CRPA) in clinical specimens from the Mulago Hospital ICU. Also, carriage of carbapenemase encoding genes and class 1 integrons was investigated, as well as mobility potential of the genetic elements among CRPA and CRAB isolates.

## Methods

### Study design and setting

This was a cross sectional study conducted at Mulago National Referral Hospital in Kampala, Uganda. Culturing and other laboratory procedures were performed at the laboratories of the Departments of Medical Microbiology and Immunology & Molecular Biology, Makerere University College of Health Sciences.

### Samples

The bacteria investigated were isolated from clinical specimens referred by physicians for diagnostic testing (mainly culturing and identification of bacterial pathogens) in the period between 2015 and 2017. During this period, the Clinical Microbiology Laboratory processed clinical specimens from a total of 9269 patients for isolation of CRAB and CRPA. The specimens included blood, tracheal aspirates, ear swabs, catheter tips, sputum, pus swabs, throat swabs etc. Majority (78%) of the specimens yielding CRAB and CRPA isolates were from the ICU while 20% were from the Ear Nose & Throat (ENT) department. The Burns unit contributed the remaining 2% of the isolates.

### Culturing, identification and drug susceptibility testing

Culturing and identification of isolates to species level was performed as described previously [1]. Briefly, presumptive identification of the isolates was based on colony morphology, Gram staining characteristics and biochemical properties. Biochemical tests included triple sugar iron (TSI), Sulphur indole motility test (SIM), as well as citrate, urease and oxidase tests. *Pseudomonas aeruginosa* colonies were identified by their spreading pattern, serrated edges, fruity sweet-grape smell and bright green color. In addition, a negative TSI, positive catalase and oxidase tests and growth at 42 °C were also used to identify *Pseudomonas aeruginosa*. *Acinetobacter* isolates were identified based on negative oxidase and glucose fermentation tests, negative catalase and motility tests and their inability to grow under anaerobic conditions. Further, to identify *Acinetobacter baumannii*, PCR-detection of the *bla*_OXA-51_ gene intrinsic to this species was performed [1, 6, 8].

Following isolate identification, drug susceptibility testing was performed using the Kirby Bauer disk diffusion method on Mueller Hinton agar (MHA) (BiolabZrt Budapest, Hungary), as recommended by the Clinical Laboratory Standards Institute (CLSI, 2014) and elsewhere [1, 9, 10]. Briefly, three colonies of the test isolate were emulsified into sterile saline and the resulting suspension adjusted to turbidity of 0.5 McFarland. Antibiotic discs (BiolabZrt Budapest, Hungary) screened against the isolates were imipenem (10 μg), meropenem (10 μg), piperacillin/tazobactam (100 μg /10 μg), colistin (10 μg), gentamicin (10 μg), amikacin (30 μg), ciprofloxacin (5 μg), cefepime (30 μg), and ceftazidime (30 μg). Interpretation of susceptibility testing results was according to CLSI guidelines (2014). Quality control procedures were done using *Pseudomonas aeruginosa* ATCC 27853 and *Escherichia coli* ATCC 35218/25922 [1, 9, 10].

### Carbapenemase assays

Phenotypic screening for carbapenemase activity was done using the Modified Hodge Test (MHT). Additionally, combination disc tests, imipenem-EDTA and boronic acid inhibition tests, were used to screen for MBL and KPC production respectively, as described previously [1, 9, 11]. In the MHT assay, a 1:10 dilution of the indicator strain (*Klebsiella pneumoniae* ATCC 700603 or *E. coli* ATCC 25922) was made by diluting 0.5 ml of culture (0.5 McFarland) to 5 ml with sterile normal saline. Then, a lawn culture (1,10 dilution) was streaked onto a MHA plate using a sterile swab. A 10 μg meropenem disk was placed in the center of the test area on the MHA plate, and test isolates streaked in a straight line from the edge of the disk to the edge of the plate. *Klebsiella pneumoniae* ATCC BAA-1705 and *Klebsiella pneumoniae* ATCC BAA-1706 were used as positive and negative controls, respectively [1, 12–14]. Furthermore, isolates that were found to be non-susceptible to imipenem or meropenem were also screened for MBL activity. In the MBL assay, overnight liquid cultures of the test isolates (adjusted to a 0.5 McFarland) were streaked on a MHA plate. Two discs of imipenem (10 μg) were placed 15 mm apart (center-to-center); of the two discs, one was impregnated with 10 μl of 0.5 M EDTA to achieve a disc content of 1.5 mg EDTA. Following this, incubation was done at 37 °C, overnight. An increase in inhibition zone diameter by ≥5 mm in the EDTA-supplemented disc was interpreted as positive for MBL production [1]. Isolates that were positive were also screened for KPC production. In this test, a 10 μg meropenem disc and another 10 μg meropenem disc containing 3- AminoPhenyl boronic Acid (3-APBA) (300 μg/ml) (Tokyo Chemical Co. Ltd., Japan) were placed 20 mm apart, center-to-center. An increase in inhibition zone diameter by ≥5 mm around the combination disc compared to the meropenem disc alone was considered positive for KPC production [11].

### Detection of carbapenemase genes and class 1 integrons

Molecular assays to determine carriage of carbapenemase encoding genes among CRAB and CRPA were performed as described previously [1, 15, 16]. The carbapenemase genes screened for included MBL encoding genes (*bla*_IMP_*, bla*_VIM_*, bla*_SPM_*, bla*_NDM_ & *bla*_KPC_) and OXA-type genes (*bla*_OXA-23_, *bla*_OXA-24_ & *bla*_OXA-58_). Primers used in PCRs and amplicon analysis were described previously [1, 17].

### Conjugation and identification of transconjugants

Conjugation experiments were performed as described previously [18, 19] using CRAB/CRPA as donors and *E. coli* J53 (F− met pro Azi^r^) which is resistant to sodium azide, as a recipient. Additional donor characteristics included co-carriage of class 1 integrons and *bla*_VIM_ gene. CRBA/CRPA (donor) and *E. coli* J53 (recipient) were mixed in a ratio of 10:1 (donor:recipient) in Luria Bertani broth and the mixture incubated at 37 °C overnight. To select for transconjugants, serial dilutions of the cultures were plated on MacConkey agar containing ceftazidime (4 μg/ml) and sodium azide (100 μg/ml), which medium is toxic to donor cells. Confirmation of transfer was determined by PCR as described above to detect *bla*_VIM_ among transconjugants.

### Quality control

Well-characterized carbapenemase producing clinical strains of *Klebsiella pneumoniae* (Nr.8) and *Pseudomonas aeruginosa* from our collection [1, 9] were used as positive controls in molecular assays. For each gene that was PCR- amplified, PCR products were randomly selected and sequenced, and sequences confirmed through BLAST-searching at the National Centre for Biotechnology Information (https://blast.ncbi.nlm.nih.gov/Blast.cgi) as previously described [1]. ‘No template’ PCR controls and negative control PCRs with template DNA extracted from carbapenemase gene negative strains (*K. pneumoniae* DSMZ 9377, *E. coli* ATCC 25922 and *P. aeruginosa* ATCC 27853) were also included [1].

## Results

A total of 9269 specimens from various wards at Mulago Hospital yielded 1077 and 488 isolates of *Acinetobacter baumannii* and *Pseudomonas aeruginosa*, respectively. Altogether, 2.7% (29/1077) and 7.4% (36/488) isolates were confirmed to be CRAB and CRPA respectively, Table 1. Acinetobacter species were detected in virtually all the tracheal aspirate samples and most of these samples yielded poly-microbial isolates that included *Klebsiella pneumoniae* and *Escherichia coli*.

**Table 1:** Clinical specimens from which CRAB and CRPA were recovered

Specimen type	No. of specimens	*Acinetobacter* species			*Pseudomonas aeruginosa*		
		No. isolated (%)	No. CRAB reported (%)	No. CRAB recovered	No. isolated (%)	No. CRPA reported (%)	No. CRPA recovered
Blood	7343	412 (5.6)	4 (0.97)	2	127 (1.72)	0	0
Sputum	908	119 (13.1)	1 (0.84)	1	73 (8.03)	7 (9.6)	4
Pus swab	350	7 (2)	0	0	5 (1.2)	4 (80)	4
Tracheal aspirate	221	344 (155.7)	11 (3.2)	10	207 (93.7)	15 (7.2)	10
Ear swab	177	117 (66.1)	7 (5.98)	4	13 (7.3)	2 (15.4)	2
Throat swab	160	13 (8.1)	3 (23)	1	13 (8.1)	3 (23.1)	1
Catheter tip	94	58 (61.7)	1 (1.7)	1	45 (47.9)	5 (8.9)	4
BAL fluid	16	7 (43.8)	2 (28.6)	2	5 (41.3)	0	0
Total	9269	1077 (11.6)	29 (2.7)	21	488 (5.3)	36 (7.4)	25

*BAL* Broncho-alveolar lavage fluid, *CRAB* Carbapenem resistant *Acinetobacter baumannii*, *CRPA* Carbapenem resistant *Pseudomonas aeruginosa*

### CRAB and CRPA isolates recovered per year and hospital ward

The CRPA isolates recovered in 2015, 2016 and 2017 were 8/21 (38%), 4/21 (19%) and 9/21 (43%), respectively: The yield per ward (ICU & ENT) was five & three isolates (2015); three & one isolate (2016); eight & one isolate (2017), respectively. Furthermore, the CRAB isolates recovered in 2015, 2016 & 2017 were 6/25 (24%), 3/25 (12%) and 16/25 (64%), respectively: The yield per ward was four & two in 2015 from ICU & ENT, respectively; three in 2016 from ICU; 13, two & one in 2017 from ICU, ENT & Burns Unit, respectively.

### Carbapenemase activity among CRAB and CRPA isolates

Carbapenemase assays performed with the MHT assay showed that 40% (10/46) and 33% (15/46) of CRPA and CRAB isolates respectively, were positive for carbapenemase production. Furthermore, 72% (18/46) and 48% (10/46) of CRPA and CRAB isolates respectively, were MBL producers and none was positive for KPC, Table 2.

**Table 2:** Carbapenemase activity among CRPA and CRAP isolates (*n* = 46)

MHT	MHT (%)		MBL activity	EDTA-DST (%)		IMP hydrolysis (%)		MEM hydrolysis (%)		KPC (*n* = 28)	KPC (%)	
	+ve	-ve		+ve	-ve	+ve	-ve	+ve	-ve		+ve	-ve
CRPA (25)	10 (40)	15 (60)	CRPA (25)	18 (72)	7 (28)	25 (100)	0	21 (84)	4 (16)	CRPA (10)	0	0
CRAB (21)	7 (33)	14 (67)	CRAB (21)	10 (48)	11 (52)	21 (100)	0	21 (100)	0	CRAB (18)	0	0
Total: 46	17	29	46	28	18	46		42	4	28	0	0

*CRPA* Carbapenem resistant *Pseudomonas aeruginosa*, *CRAB* Carbapenem resistant *Acinetobacter baumanii*, *EDTA-DST* EDTA disk synergy test, *IMP* Imipenem, *MEM* Meropenem, *KPC Klebsiella pneumoniae* carbapenemase, *+ve* Positive, *−ve* Negative

### Prevalence of carbapenemase genes and class 1 integrons

*bla*_VIM_ was the most prevalent carbapenemase gene and it was detected in all CRPA and CRAB isolates, Table 3. Of note, the other MBL encoding genes i.e. *bla*_IMP_, *bla*_SPM,_ and *bla*_NDM_, were not detected; as well, carriage of the OXA-type carbapenemase genes was not detected in CRPA. Also, in agreement with activity assays, none of our isolates was positive for *bla*_KPC_ carriage. We however found the OXA-type carbapenemase genes to be prevalent among CRAB i.e. 29% & 24% for *bla*_OXA-23_ & *bla*_OXA-24_, respectively. Co-carriage of *bla*_OXA-23_ and *bla*_OXA-24_ was seen in 14% of the CRAB isolates. All CRAB isolates carried the *bla*_OXA-51_ gene while none carried *bla*_OXA-58_. Class 1 integrons of sizes 500 bp to 1000 bp were detected in 63% (29/46) of the isolates: 64% (16/25) & 62% (13/21) CRPA & CRAB respectively, Table 3. Furthermore, the classification of *Acinetobacter baumanii* based on *bla*_OXA-51_ gene sequences [20, 21] showed that 12, eight and one CRAB isolate in this study belonged to pan-European International Clones II, I & III, respectively.

**Table 3:** Prevalence of class 1 integrons among *bla*_VIM_-positive isolates

No. screened	No. (%) with *bla*_VIM_		No. (%) with *bla*_VIM_ + class 1 integron	
	Positive	Negative	Positive	Negative
CRPA (25)	25 (100)	0 (0)	16 (64)	9 (36)
CRAB (21)	21 (100)	0 (0)	13 (62)	8 (38)

All the *bla*_OXA-23_-positive isolates were detected in samples from the ICU; these were tracheal aspirates and sputum samples -tracheal aspirates provided 83% of *bla*_OXA-23_ positive CRAB while sputum samples yielded 17%. Likewise, 80% of *bla*_OXA-24_ positive CRAB isolates were detected in tracheal aspirates from the ICU. All isolates with co-carriage of *bla*_OXA-23_ and *bla*_OXA-24_ genes were detected in tracheal aspirates from the ICU. Only 20% of the *bla*_OXA-24_ positive CRAB isolates were from the ENT ward.

### Drug susceptibility profiles of CRAB and CRPA isolates

*bla*_VIM_-plus-*bla*_OXA_ positive CRAB exhibited high levels of resistance to commonly used antibiotics especially ceftazidime (100%), gentamicin (88%), ciprofloxacin (88%), cefepime (75%), piperacillin/tazobactam (63%), and amikacin (50%), Table 4. Most of the CRAB and CRPA isolates were MDR (resistance to three or more classes of antimicrobials) however, all were susceptible to colistin. Furthermore, the susceptibility profiles of isolates carrying a single OXA-type gene (either *bla*_OXA-23_ or *bla*_OXA-24_) were not different from profiles of isolates that carried two genes concurrently.

**Table 4:** Susceptibility profiles of CRAB carrying both *bla*_OXA-23_-like and *bla*_OXA-24_-like genes

Isolate No.	Year of isolation	IMP	MEM	CN	PTZ	CAZ	AK	FEP	CIP	CT
4268	2015	R	R	R	R	R	R	R	R	S
2932	2015	R	R	R	R	R	R	S	R	S
4643	2015	R	R	R	I	R	S	I	R	S
1566.3	2015	R	R	S	R	R	S	R	R	S
1566	2016	R	R	R	I	R	S	R	I	S
2753.1	2016	R	R	R	I	R	I	R	R	S
4264.2	2017	R	R	R	R	R	R	R	R	S
4264.1	2017	R	R	R	R	R	R	R	R	S
% R		100	100	88	63	100	50	75	88	0

*IMP* Imipenem, *MEM* Meropenem, *CN* Gentamicin, *PTZ* Piperacillin/tazobactam, *CAZ* Ceftazidime, *AK* Amikacin, *FEP* Cefepime, *CIP* Ciprofloxacin, *CT* Colistin, *R* Resistant, *I* Intermediate, *S* Susceptible. 4264.2, 4268 & 4264.1 carried both *bla*_OXA-23-like_ & *bla*_OXA-24-like_ genes

Similarly, *bla*_VIM_-positive CRPA isolates showed high level resistance to gentamicin (100%), ceftazidime (89%), amikacin (72%), cefepime (72%), piperacillin/tazobactam (56%) and ciprofloxacin (50%); 94% of the isolates were MDR but susceptible to colistin. As well, all CRAB and CRPA isolates that were positive for both *bla*_VIM_ and class 1 integrons were highly resistant to the tested antibiotics but the susceptibility profiles were not different from isolates that did not carry class 1 integrons, Table 5.

**Table 5:** Susceptibility profiles of CRAB and CRPA positive for *bla*_VIM_ plus class 1 integrons

Isolate No.	Year of isolation	Carbapenem resistant *Acinetobacter baumanii*								
		IMP	MEM	CN	PTZ	CAZ	AK	FEP	CIP	CT
4268^a^	2015	R	R	R	R	R	R	R	R	S
2932^a^	2015	R	R	R	R	R	R	S	R	S
4643^a^	2015	R	R	R	I	R	S	I	R	S
1566.3^a^	2015	R	R	S	R	R	S	R	R	S
2932.2^a^	2015	R	R	I	I	R	S	S	R	S
4722.1	2015	R	R	I	I	R	S	R	I	S
5730.2^a^	2015	R	R	R	I	R	R	R	I	S
1566^a^	2016	R	R	R	I	R	S	R	I	S
2753.1	2016	R	R	R	I	R	I	R	R	S
1688^a^	2016	R	R	R	I	R	S	I	I	S
2026.2	2016	R	R	R	S	I	S	I	I	S
4264.2^a^	2017	R	R	R	R	R	R	R	R	S
4264.1^a^	2017	R	R	R	R	R	R	R	R	S
4725.1	2017	R	R	R	I	R	S	I	R	S
2987.1^a^	2017	R	R	R	I	I	S	S	S	S
3523	2017	R	R	R	R	R	S	R	R	S
4725.2	2017	R	R	R	R	R	R	R	R	S
1314.1	2017	R	R	R	I	R	R	R	R	S
1314.2	2017	R	R	S	I	R	S	R	R	S
% R^a^		100	100	82	46	91	46	55	55	0
% R		100	100	79	37	90	42	63	63	0
Carbapenem resistant *Pseudomonas aeruginosa*										
4786	2015	R	R	R	I	R	R	R	I	S
5730^a^	2015	R	R	R	I	R	R	R	I	S
3361^a^	2015	R	R	R	R	R	R	R	R	S
0620^a^	2015	R	R	R	I	R	S	S	I	S
6797^a^	2016	R	R	R	R	R	R	I	R	S
1688.2^a^	2016	R	R	R	I	R	R	R	I	S
0004.1	2017	R	R	R	R	R	R	R	I	S
3512^a^	2017	R	I	R	R	S	R	R	R	S
3168	2017	R	R	R	I	R	S	R	I	S
1864	2017	R	R	R	R	R	R	I	I	S
0466.2^a^	2017	R	R	R	R	R	R	R	R	S
0004.2^a^	2017	R	R	R	I	R	R	R	R	S
0466.1^a^	2017	R	R	R	R	R	R	R	R	S
0903^a^	2017	R	R	R	R	R	S	R	R	S
3337.1^a^	2017	R	R	R	R	R	R	R	R	S
3337.2^a^	2017	R	R	R	R	R	R	R	R	S
0921^a^	2017	R	R	R	I	I	S	S	S	S
3476	2017	R	R	R	I	R	S	S	I	S
% R^a^		100	92	100	62	85	77	77	69	0
% R		100	94	100	56	89	72	72	50	0

^a^*bla*_VIM_ plus class 1 integron positive isolates; *IMP* Imipenem, *MEM* Meropenem, *CN* Gentamicin, *PTZ* Piperacillin/tazobactam, *CAZ* Ceftazidime, *AK* Amikacin, *FEP* Cefepime, *CIP* Ciprofloxacin, *CT* Colistin, *R* Resistant, *I* Intermediate, *S* Susceptible

### Transfer experiments

To demonstrate the potential for mobility of carbapenemase encoding genes in the hospital environment, conjugation experiments were successfully performed. Donors were CRPA and CRAB isolates that were positive for both *bla*_VIM_ and class 1 integrons while the recipient was sodium azide resistant *E. coli* strain J53*.* PCR assays showed that 25 and 39% of CRPA and CRAB donors respectively, successfully transferred the *bla*_VIM_ genetic element to *E. coli*, Additional file 1: Figure S1.

## Discussion

Antimicrobial drug resistance (AMR) is a well-recognized global problem and high rates of resistance to first-line antibiotics among Gram-negative bacteria have been reported in Uganda [22]. As such, routine surveillance studies are necessary to monitor antibiotic resistance trends in low-income countries where antibiotic selection pressure is likely to be high.

This study has revealed that the prevalence of CRAB and CRPA in clinical specimens from Mulago Hospital remains relatively low and comparable to recently reported rates for carbapenem resistant Gram negative bacteria in Uganda [1, 9, 23]. Importantly, almost all the CRAB and CRPA isolates were from the ICU. Furthermore, *bla*_VIM_ and *bla*_OXA-23_ were the most prevalent carbapenemase genes with the former being detected in all CRAB and CRPA isolates. As such, *bla*_VIM_ and *bla*_OXA-23_ are the genes mediating carbapenem resistance in CRAB and CRPA in the Mulago Hospital ICU. Again, these findings are consistent with a recent study at Mulago Hospital and other settings in developing countries where similar studies have been done [1–3, 9, 24]. In addition to the *bla*_VIM_ gene, we also detected *bla*_OXA-23_, *bla*_OXA-24_ and *bla*_OXA-51_ genes in CRAB. Unlike *bla*_OXA-23_- and *bla*_OXA-24_-like genes, *bla*_OXA-51_ naturally occurs in *Acinetobacter baumannii* [1, 6, 8]. Furthermore, while in the previous study at Mulago [1] the detection of *bla*_OXA-58_-like gene in CRAB was reported, it was not detected in this study. However, this observation is in agreement with studies by Minandri et al. 2011 [25] and Wu et al. 2015 [26] who discussed the transition from carriage of *bla*_OXA-58_-like to *bla*_OXA-23_-like and *bla*_OXA-24_-like genes. Furthermore, through conjugation experiments we demonstrated the potential for horizontal gene transfer of the *bla*_VIM_ gene. Transfer rates in our study were low but in agreement with another study [27] which reported similar transfer rates. We have therefore provided evidence suggestive of mobility for carbapenem resistance genes in the Mulago hospital environment via horizontal gene transfer processes.

Although CRAB and CRPA that simultaneously carried *bla*_VIM_ and class 1 integron were highly resistant to antibiotics, the susceptibility profiles were not different from isolates that did not carry class 1 integrons. Hence, carriage of integrons did not affect the resistance profiles implying that integrons in these isolates either did not carry drug resistance genes or they carried cassettes encoding resistance phenotypes that were not screened for. Furthermore, the susceptibility profiles of CRAB carrying a single OXA-type gene (either *bla*_OXA-23_ or *bla*_OXA-24_) were not different from profiles of isolates that carried two genes concurrently. As *OXA* genes generally confer a β-lactam resistance phenotype, the additive effect might be better detected with minimum inhibition concentrations (MICs), not agar diffusion approaches we used.

Additionally, although the CRAB and CRPA isolates we investigated were not associated with outbreaks, their detection in clinical specimens is of public health significance. Indeed, we observed high resistance to common antibiotics but this has been reported before [1, 2, 26]. Of note, carriage of *bla*_OXA-23_ alone, or *bla*_OXA-23_ plus *bla*_OXA-24_ in bacteria has been associated with high resistance to carbapenems and to other classes of antimicrobials [25, 26]. While *bla*_VIM_ carriage in this setting has been reported before [3, 28, 29], a high prevalence of *bla*_VIM_ is worrying as this genetic element renders carbapenems (imipenem & meropenem) ineffective [3, 29–31], narrowing treatment options to colistin & polymyxin B that have been associated with drastic side effects e.g. nephrotoxicity, neurotoxicity, etc. [3, 29–31]. As such, a narrow window remains before total antimicrobial resistance is seen in these bacterial isolates as they continue to be subjected to antibiotic pressure in the hospital [32–35]. Unlike in the previous study [1] we did not detect the three MBL genes *bla*_SPM_, *bla*_NDM_ *and bla*_IMP_ that have been associated with high resistance to carbapenems especially to imipenem [36, 37]. Further, the high prevalence of *bla*_VIM_ in CRAB and CRPA in this study is suggestive of the role of the accessory genome in evolution of carbapenem resistance in our setting. *bla*_VIM_ is characterized as an acquired MBL gene translocated between bacteria via class 1 integrons, in horizontal gene transfer mechanisms mostly involving conjugative plasmids [28, 38–40].

There were certain limitations in this study. We screened isolates for colistin susceptibility using the agar diffusion method instead of the recommended broth microdilution or the Etest methods. Further studies should take this into consideration given that poor agar dilution characteristics can limit the predictive accuracy of the disk diffusion method.

## Conclusions

*bla*_VIM_ and *bla*_OXA-23_ are the most prevalent carbapenemase genes among CRAB and CRPA isolates at the Mulago Hospital ICU. There is need to improve infection control practices at the hospital to curb the transmission of the isolates in the hospital.

## Supplementary information

**Additional file 1:** **Figure S1.** A representative image showing donors and trans-conjugants screened for presence of *bla*_VIM_ gene. Lanes: L, 100 bp DNA ladder; D, 382 bp *bla*_VIM_ fragment in donors (CRAB or CRPA isolate); T, 382 bp *bla*_VIM_ fragment in trans-conjugant (*E. coli* J53, recipient); P, *bla*_VIM_ carrying strain (positive control); J53, unconjugated *E. coli* J53 (negative control recipient).

## Data Availability

The datasets used and/or analyzed during the current study are available from the corresponding author on reasonable request.

## Abbreviations

ATCC: American Type Culture Collection
*bla*: β-lactamase
BLAST: Basic Local Alignment Search Tool
CLSI: Clinical laboratory Standards Institute
CRAB: Carbapenem Resistant *Acinetobacter baumannii*
CRPA: Carbapenem Resistant *Pseudomonas aeruginosa*
EDTA: Ethylene Diamine Tetra Acetic acid
ENT: Ear, Nose and Throat
ICU: Intensive Care Unit
IMP: Imipenemase
KPC: *Klebsiella pneumoniae* carbapenemase
MBL: Metallo-β-lactamase
MHA: Mueller Hinton Agar
MHT: Modified Hodges Test
NDM: New Delhi Metallo- β-lactamase
OXA: Oxacillinase
PCR: Polymerase Chain Reaction
SIM: Sulphur Indole Motility
SPM: Sao Paulo Metallo- β-lactamase
TSI: Triple Sugar Iron
VIM: Verona integron encoded Metallo-β-lactamase
