# Supplementary figures and images for: blaVIM- and blaOXA-mediated carbapenem resistance among Acinetobacter baumannii and Pseudomonas aeruginosa isolates from the Mulago hospital intensive care unit in Kampala, Uganda
Source: BMC Infect Dis. 2019 Oct 16;19:853. doi: 10.1186/s12879-019-4510-5 (PMC6794873; doi:10.1186/s12879-019-4510-5)

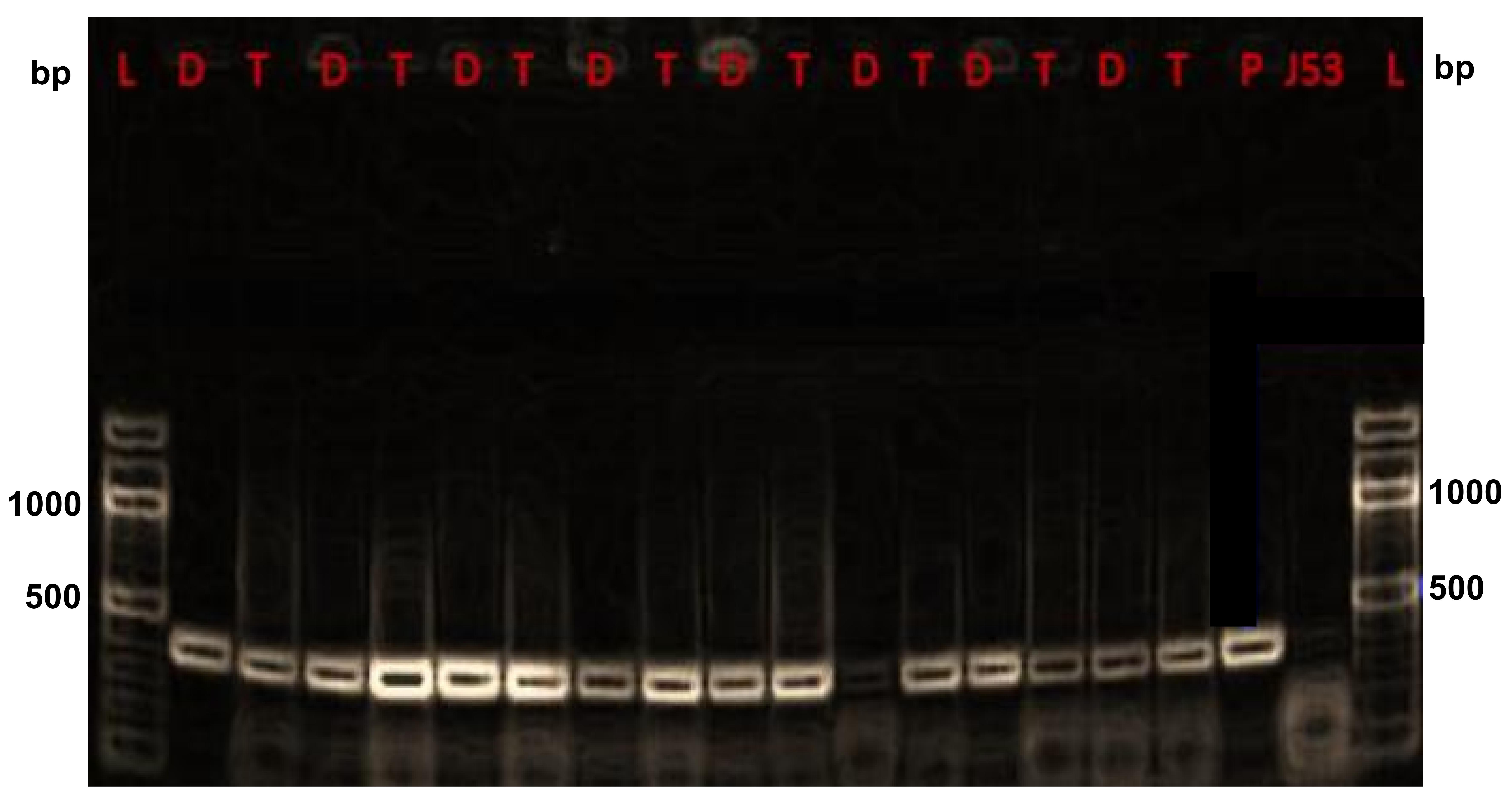

Supplement: Supplementary file 1 — Additional file 1: Figure S1. A representative image showing donors and trans-conjugants screened for presence of blaVIM gene. Lanes: L, 100 bp DNA ladder; D, 382 bp blaVIM fragment in donors (CRAB or CRPA isolate); T, 382 bp blaVIM fragment in trans-conjugant (E. coli J53, recipient); P, blaVIM carrying strain (positive control); J53, unconjugated E. coli J53 (negative control recipient). [file 12879_2019_4510_MOESM1_ESM.tiff]
